# Tune Up In Situ Autovaccination against Solid Tumors with Oncolytic Viruses

**DOI:** 10.3390/cancers10060171

**Published:** 2018-05-31

**Authors:** Teresa Nguyen, Naze G. Avci, Dong Ho Shin, Naiara Martinez-Velez, Hong Jiang

**Affiliations:** 1 Department of Neuro-Oncology, The University of Texas MD Anderson Cancer Center, 6767 Bertner St., Houston, TX 77030, USA; tnguyen36@mdanderson.org (T.N.); dshin4@mdanderson.org (D.H.S.); 2 Neurosurgery Research, Houston Methodist Research Institute, Houston, TX 77030, USA; ngavci@houstonmethodist.org; 3 Pediatric Department, Clínica Universidad de Navarra, 31008 Pamplona, Spain; nmartinez.20@alumni.unav.es

**Keywords:** oncolytic virus, in situ autovaccination, cytokine, immune checkpoint inhibitor, immune co-stimulator

## Abstract

With the progress of immunotherapy in cancer, oncolytic viruses (OVs) have attracted more and more attention during the past decade. Due to their cancer-selective and immunogenic properties, OVs are considered ideal candidates to be combined with immunotherapy to increase both specificity and efficacy in cancer treatment. OVs preferentially replicate in and lyse cancer cells, resulting in in situ autovaccination leading to adaptive anti-virus and anti-tumor immunity. The main challenge in OV approaches is how to redirect the host immunity from anti-virus to anti-tumor and optimize the clinical outcome of cancer patients. Here, we summarize the conceptual updates on oncolytic virotherapy and immunotherapy in cancer, and the development of strategies to enhance the virus-mediated anti-tumor immune response, including: (1) arm OVs with cytokines to modulate innate and adaptive immunity; (2) combining OVs with immune checkpoint inhibitors to release T cell inhibition; (3) combining OVs with immune co-stimulators to enhance T cell activation. Future studies need to be enforced on developing strategies to augment the systemic effect on metastasized tumors.

## 1. Introduction

The history of cancer therapy is a witness of toxicity and failure of efficacy despite numerous efforts to identify druggable cancer targets for personalized and targeted treatments. Emerging evidence indicates that the main challenges in developing efficacious and safe cancer therapeutics are heterogeneity, even within a single cancer, and the evolution of cancer cells during therapy [1,2]. Thus, it is imperative to develop novel strategies to overcome these obstacles.

The immune system is capable of initiating effective responses specifically toward certain molecular targets, such as antigens from pathogens. This type of reaction would be promising for cancer therapy if it could be redirected efficiently against all cancer cell populations. To modulate the immunity against cancers, some pro-inflammatory cytokines, including interleukin-2 (IL-2), interleukin-12 (IL-12), tumor necrosis factor (TNF), and interferon (IFN), have been used to treat malignancies [3,4]. However, the systemic administration of these agents often leads to dose-dependent side-effects (e.g., hypotension, flu-like symptoms, nausea, capillary leak), preventing the escalation to doses that are therapeutically active [4]. During the past two decades, immune checkpoint blockade profoundly changed cancer immunotherapy [5]. Clinical studies have demonstrated the efficacy of these types of therapies in a variety of malignancies, although it is more effective in cancers with an immunogenic tumor microenvironment compared to those with a non-immunogenic microenvironment [5,6]. Though to a less extent, like cytokine therapy, immune checkpoint blockade can cause immune-related adverse events (irAEs) in many patients due to the overstimulation of immune reactivity that may result in autoimmunity [7]. Moreover, to increase the efficacy in patients who are refractory to single antibody blockade, different immune checkpoint blocking antibodies have been combined to treat these patients [8,9]. This may unavoidably increase the risk for irAEs.

To pursue the specificity and safety of immunotherapy, efforts have been made to define cancer-associated antigens and develop therapeutic cancer vaccines. Currently, therapeutic cancer vaccination is only effective as monotherapy for the treatment of premalignant or minimal residual disease, but not in established cancers [10]. Vaccine strategies can increase the frequency and activity of tumor-specific T cells. However, they have failed to ensure that these T cells could infiltrate into tumors and/or exert their function within the tumor due to the immunosuppression in the tumor [10]. Moreover, since cancer vaccines only target a limited number of antigens in the cancer antigen repertoire, after immune editing during the therapy, cancer cells without expression of these antigens can escape and give rise to new tumor cell populations that are resistant to the same vaccine therapy [10,11].

Oncolytic viruses (OVs) are genetically modified or naturally occurring viruses that selectively replicate in and disrupt cancer cells [12,13,14]. Theoretically, these viruses can cause a cascading oncolytic effect in the entire tumor [12,13,14], resulting in the eradication of the infected tumors. However, the viruses have hardly reached their full therapeutic potential due to the antiviral immune responses of the patients and the dynamic immune suppression within the tumor environment [12,13,14]. Nevertheless, OVs have been clinically demonstrated to initiate systemic antitumor immunity due to the in situ cancer vaccination effect of the therapy [12,13,14]. That is, during virotherapy, the in situ viral infection, replication, and subsequent tumor necrosis cooperate to disrupt immunosuppression within the tumor microenvironment, resulting in T cell reactivity against cancer neo-antigens [15,16,17]. Taken together, it seems there could be an opportunity to take advantage of the above strategies to disrupt the immunosuppression within the tumor, upregulate the activity of tumor-specific T cell, and thus further develop more efficacious and safe therapies for cancer patients.

## 2. In Situ Autovaccination against Cancers Induced by Oncolytic Viruses

Immunity is a double-edged sword in cancer therapy. Thus, it is as important to steer the immune response specifically to cancer cells as to disrupt them efficiently. Unlike cytokine therapy or immune checkpoint blockade that modulate the whole population of certain types of immune cells, cancer vaccines induce immunity specifically against cancer cells. However, in addition to its inefficiency in established cancers [10], this strategy is also challenged by the identification of universal tumor-associated antigens (TAAs) and the difficulty in isolating and preparing individualized vaccines ex vivo [18,19]. During the process of initiation and progression, cancers acquire tens to thousands of nonsynonymous mutations [20,21]. These mutations (either driver or passenger) result in changes of the amino acid sequence or protein structure to produce neo-antigens [22,23]. Since these antigens are not expressed by normal cells, they are predicted to be recognizable by the immune system and be specific targets of immunotherapy. Thus, to improve the effectiveness of cancer vaccine therapy, immunomodulatory agents have been delivered directly into tumors to cause an in situ autovaccination effect. It enhances the immunogenicity of the treated tumor, generates tumor infiltrating lymphocytes (TILs), and triggers a systemic anti-tumor immune response [19]. 

Accumulating evidence demonstrates that the efficacy of many OVs is at least partly related to the induction of potent antitumor immunity as a result of the in situ vaccination effect of the treatment [12]. When OVs are delivered intratumorally, the infection gives rise to pathogen-associated molecular patterns (PAMPs) [24]. In addition, the replication of the viruses and the consequential lysis of the infected cells, a type of immunogenic cell death, release damage- (or danger-) associated molecular patterns (DAMPs) [17,25,26]. These molecules can be recognized by cells of the innate immune system to initiate an inflammatory immune response [24,27,28]. Thus, the immune suppressive tumor microenvironment is changed to an immune active one, increasing the infiltration of immune cells to the tumor site [16,17]. The tumor-associated antigens (TAAs) from virally lysed cancer cells are released to the tumor milieu and are then cross-presented to T cells by endogenous antigen-presenting cells (APCs), including dendritic cells (DCs) and macrophages. Moreover, tumor cells with or without viral infection can also function as APCs to present TAAs to T cells. In our studies, oncolytic adenoviruses induce autophagy leading to immunogenic cell death, and upregulate proteasome activity and MHC expression in the infected cells, resulting in increased presentation of viral antigens and TAAs to T cells to stimulate their activation [15,17]. The consequential adaptive immunity not only inhibits the treated tumors but also has effect on the distant disseminated tumors and results in immune memory against the same cancer cells [16,17].

**Figure 1:**
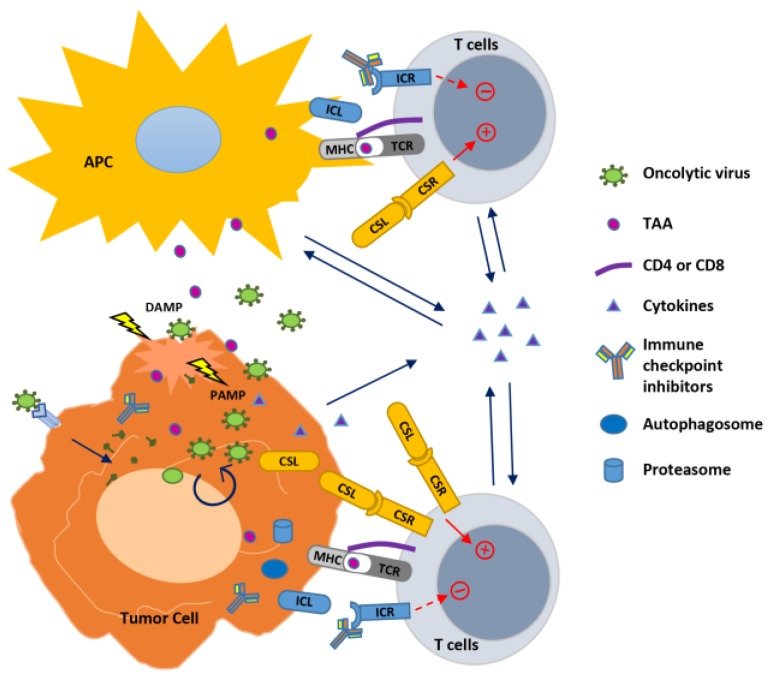
Strategies to improve oncolytic virus-mediated anti-tumor immunity. Oncolytic viruses (OVs) infect and replicate inside cancer cells, resulting in cell lysis and propagation of virions to infect nearby cancer cells. This process generates pathogen-associated molecular patterns (PAMPs) and damage- (or danger-) associated molecular patterns (DAMPs) that trigger an innate immune response to modulate the tumor microenvironment, resulting in in situ autovaccination leading to adaptive anti-virus and anti-tumor immunity. In the infected tumor cells, OVs also induce autophagy (autophagosome formation) and activity of proteasome to increase their capability to function as APC to present tumor-associated antigens (TAAs) to T cells. OVs have been combined with immune modulators to enhance immunity against the tumor. Cytokines expressed by OVs stimulate innate and adaptive immunity within the tumor. Combination of OVs with immune checkpoint blockade through antibodies to inhibit the interaction between immune checkpoint ligand (ICL) and receptor (ICR), or with agonist antibody or expression of the co-stimulatory ligand (CSL) to bind with the co-stimulatory receptor (CSR) augments T cell receptor (TCR) signaling initiated by the virus through presenting TAAs with major histocompatibility complex (MHC), leading to enhanced T cell activation against the tumor.

Results from clinical trials show that complete responses to OVs as a single agent have rarely been observed [12]. In order to achieve effective virotherapy, induction of potent and sustaining antitumor immunity is critical. During T cell development for adaptive immunity, the T Cell Receptor (TCR) signaling (Signal 1) initiates the reaction, then co-stimulation and/or co-inhibition (Signal 2) shape the outcome of T cells, and cytokines (Signal 3) determine whether Signals 1 and 2 cause tolerance or a productive response leading to potent effector functions, survival, and formation of immune memory [29,30]. Intratumoral delivery of the OVs generates an inflammatory environment in the tumor and initiates the TCR signaling through cross-presenting TAAs to T cells while the reaction is to some extent also modulated by co-signaling and cytokines. Thus, it is logical to combine the viruses with immunotherapeutic strategies to modulate the three signals to enhance the in situ and abscopal antitumor effect (Figure 1).

## 3. Strategies to Boost Oncolytic Virus-Induced Anti-Cancer Immunity

### 3.1. Oncolytic Viruses and Cytokines: Modulate the Innate and Adaptive Immune Response

The complex interaction between tumor cells and the tumor microenvironment components, including fibroblasts, extracellular matrix, blood vessels, inflammatory cells, and stimulatory molecules such as chemokines and cytokines, plays an important role in tumor development. Although the interaction mostly occurs via direct cell-cell contact, the secretion of the molecular messenger cytokines stimulates immune cell recruitment to the tumor site. Pro-inflammatory cytokines, such as granulocyte-macrophage colony-stimulating factor (GM-CSF), IL-12, IL-2 and interferons (IFNs), are highly considered for anticancer therapeutic applications [31,32,33,34]. For cancer vaccination strategies, the administration or expression of cytokines at the site of tumors has been shown to increase the efficacy of cytokine therapy and decrease the toxic side effects [35]. Thus, intratumoral delivery of the cytokines with OVs were the first to be developed to modulate anti-tumor immunity (Table 1). Some cytokine-expressing OVs have already been tested in clinical trials.

GM-CSF is secreted by many cell types including T cells, macrophages, endothelial cells, and fibroblasts in response to immune stimuli [50]. It mediates antitumor immune responses by recruiting dendritic cells and macrophages [31]. Injections of GM-CSF-secreting tumor cells increased the infiltration of professional APCs, resulting in the recognition of circulating TAAs by CD4+ and CD8+ T cells [51]. GM-CSF-expressing oncolytic virus Ad5-D24-GMCSF have been shown to mediate tumor-specific immunity in an immunocompetent syngeneic hamster model [52]. In patients with advanced solid tumors refractory to standard therapies, the virus induced immune response in injected and non-injected tumors, resulting in both tumor-specific and virus-specific immunity [52]. Another version of this GM-CSF-expressing virus Ad5/3-D24-GMCSF (ONCOS-102) containing a genetically modified fiber with a serotype 3 knob was tested in a Phase I clinical trial for patients with solid tumors refractory to available treatments [36]. The trial showed that this virus was safe and the patients were able to tolerate the tested dose. Furthermore, this virus was associated with an infiltration of CD8+ T cells and upregulation of PD-L1 within the tumor, and caused an antitumor immune response related with the clinical efficacy. [36]. In addition, a first in human Phase I clinical study of CG0070, an oncolytic adenovirus with selective E1A and GM-CSF expression in Rb pathway-defective cells [53], showed that intravesical delivery of the virus was associated with a tolerable safety profile and anti-bladder cancer activity [37]. Furthermore, a Phase II/III trial of the virus for bcg-refractory non-muscle-invasive bladder cancer demonstrated that intravesical CG0070 caused a durable response in a subset of high-risk patients and has an attractive toxicity profile [38]. 

Herpes simplex virus type 1 (HSV-1)-derived OVs have also been engineered to express GM-CSF and showed significant tumor growth inhibition in vitro using human tumor cell lines and in vivo using mouse cancer models [54,55]. Talimogene laherparepvec (T-VEC, also known as OncoVEX^GM-CSF^, Imlygic^TM^) is the first oncolytic virus to be approved for melanoma treatment by FDA in the USA in 2015 and was subsequently approved in Europe and in Australia in 2016 for clinical trials [56]. In patients with stage IIIC and IV melanoma, direct injection of T-VEC induced local and systemic antigen-specific T cell responses and decreased regulatory T cells (Tregs), suppressor T cells, and myeloid-derived suppressor cells (MDSC) in patients displaying therapeutic responses [39]. The virus was also compared with GM-CSF monotherapy in patients with unresected stage IIIB to IV melanoma in a randomized open-label Phase III trial [40]. Intratumorally administered T-VEC was well tolerated and induced significant benefits in tumor regression, a higher durable response rate, and longer median overall survival than GM-CSF monotherapy [40]. These results suggest that improved systemic immunity might potentially lead to an antitumor and antiviral T cell response and prolong the overall survival. This also underlines the necessity of OV for therapeutic efficacy [40]. Based on these observations, several clinical trials of T-VEC in combination with systemic administration of immune checkpoint inhibitors (ICIs) are ongoing.

Additionally, due to its immunogenic nature, vaccinia virus produces a strong cytotoxic T lymphocyte (CTL) response and has been used to express human GM-CSF (JX-594, also known as Pexa-Vec) [57]. Increased antitumor immune response through infiltration of CD4+ and CD8+ T cells and cancer cell-selective replication were shown in an immunocompetent liver tumor model treated with JX-594 [58]. Phase I, II and III clinical trials were carried out with JX-594 in various cancers in adult and pediatric patients [43,44,45,46,47]. The therapy was well-tolerated and resulted in efficient viral replication in metastatic tumors, which also successfully recruited adaptive immune cells at the site of infection [43,44,45]. A paramyxoviruses family member, Newcastle disease virus (NDV), is one of the naturally occurring viruses with inherent oncolytic ability, and was investigated as novel cancer therapy [59,60]. Recombinant NDVs with an inserted GM-CSF gene increased the stimulation of human peripheral blood mononuclear cells (PBMC) by the infected tumor cells and led to a much higher IFN-α production in these cells compared to the control virus with no transgene, suggesting that GM-CSF-armed NDV could be a potential tumor immunotherapy to enhance immune cell infiltration [61].

IL-12 is produced by phagocytic cells and antigen-presenting cells in response to antigenic stimulation [62]. IL-12 targets NK cells, T cells, DCs, and macrophages, and stimulates the production of IFN-γ [62]. It also mediates T helper type 1 (Th1) differentiation and enhances the cytolytic effect of NK cells and CTLs [62]. IL-12 represents a potential candidate for tumor immunotherapy in murine models of melanoma, colon carcinoma, mammary carcinoma, and sarcoma [63,64,65,66,67,68]. To avoid the clinical toxicity and side effects, OVs offer a promising platform for the delivery of IL-12, restricting its expression within the tumor microenvironment. Several adenoviral vectors expressing IL-12 have been studied to investigate the regression of malignant tumors. The studies in a mouse model of mammary adenocarcinoma demonstrated that tumor regression was mediated through the induction of specific antitumor antigen CTLs which secreted IFN-γ [69]. IL-12 co-expressing B7-1 (YKL-IL12/B7), IL-18 (RdB/IL-12/IL-18), and 4-1BBL oncolytic adenoviruses have been generated to evaluate the antitumor effect of the virus in a murine melanoma B16-F10 tumor model [70,71,72]. The antitumor immunity was shown to be associated with increase of Th1/Th2 cytokine ratio and upregulation of IL-12, IL-18, IFN-γ, and GM-CSF within the tumor tissues [70,71,72]. These results suggest that an antitumor immune response is potentially mediated by the increased antitumor CTLs and IFN-γ-releasing immune cells. The intratumoral administration of oncolytic adenovirus co-expressing IL-23 and p35, the subunit of IL-12, stimulated an antitumor effect in a murine B16-F10 syngeneic tumor model, by inducing the up-regulation of IL-12, IL-23, IFN-γ, and TNF-α within the tumor tissues and reducing the Tregs frequency [73]. Oncolytic adenovirus Ad5-yCD/mutTKSR39rep-mIL12, which expresses two suicide genes and IL-12, induced high levels of IL-12 and IFN-γ in serum and tumor, increased natural killer (NK) and CTL lytic activities, and the developed tumor-specific antitumor immunity in prostate adenocarcinoma model, resulting in a significant increase in survival [74]. In addition, based on favorable results in murine tumor models [75,76,77], a Phase I clinical trial has been designed to evaluate the therapeutic effect of oncolytic HSV M032 expressing human IL-12 in in patients with recurrent/progressive glioblastoma multiforme, anaplastic astrocytoma, or gliosarcoma [48]. Consistently, IL-12 expression also enhanced the anti-tumor activity of oncolytic vesicular stomatitis virus [78,79].

IL-2 is secreted by activated T cells [80]. It is crucial for T cell activation, proliferation and differentiation [80]. NDV expressing human IL-2 was generated and demonstrated to express IL-2 upon infection of various human cancer cell line [81]. IL-2 expression further augmented the immunostimulatory properties of NDV to activate human T cells [82]. Intratumoral injections of recombinant NDV expressing IL-2 elicited immune reaction and induced dramatic reduction in tumor growth, resulting in complete and long-lasting remission in the mice bearing colon carcinoma or hepatoma [83,84]. NDV expressing both IL-2 and/or IL-12 was more efficient than NDV in stimulating INF-γ expression and inducing tumor regression in the murine hepatoma carcinoma model, resulting in immune memory against the same tumor rechallenge [85]. It was reported that IL-12 upregulated IL-2R alpha-chain (CD25) expression and stimulated the proliferation of Th1 clones, but not Th2 clones [86]. This explains the collaborating therapeutic effect of these two cytokines. In a small pilot clinical study, IL-2 expressing oncolytic vaccinia virus demonstrated minimal toxicity and persistent IL-2 expression up to 3 weeks post viral injection [49].

IFN-γ is primarily secreted by T lymphocytes, NK cells, and APCs [87]. It is a major stimulator of macrophages and promotes Th1 differentiation of CD4+ T cells by inhibiting Th2 cytokine production (IL-4 and IL-10) [87]. IFN-γ also enhances the expression of MHC I, MHC II [88], thus promoting antigen presentation and antigen processing and destruction of intracellular pathogens [89,90,91]. An engineered vesicular stomatitis virus (VSV) encoding the IFN-γ has been used in 4T1 mammary adenocarcinoma and other murine tumor models [92]. The virus-treated tumors showed increased activation of DCs and T cell, and slowed tumor growth [92]. This improved efficacy was lost in immunocompromised animals, suggesting the T cell-dependent mechanism of action and the role of IFN-γ in tumor immunosurveillance [92]. 

In summary, GM-CSF-armed adenovirus, vaccinia virus, and especially HSV, have been examined more intensively in clinic. T-VEC has gained approval in USA and Europe to treat advanced melanomas. IL-12-armed adenovirus and HSV have been tested in Phase I clinical trials. The OVs armed with IL-2 or IFN-γ have been mainly tested pre-clinically. 

### 3.2. Oncolytic Viruses and Immune Checkpoint Blockade: Release the “Brake” on T Cell Activation

The co-signaling (stimulatory or inhibitory) together with TCR signaling direct T cell function and determine T cell fate [29]. The inhibitory immune checkpoint molecules mediate tolerance to self-antigens and prevent auto-immunity [5]. These molecules are expressed on T cells in the tumor microenvironment and inhibit T cell function [5], including well studied cytotoxic T-lymphocyte associated protein 4 (CTLA-4, also known as CD152) [93] and programmed cell death protein 1 (PD-1, also known as CD279) [94]. 

CTLA-4 decreases T cell activation by outcompeting T cell co-stimulatory receptor CD28 in binding CD80 (also known as B7.1) and CD86 (also known as B7.2), actively delivering inhibitory signals to the T cell [5,95,96,97]. CTLA-4 blockade causes a broad enhancement of immune responses [5]. Recent clinical studies using ipilimumab, a CTLA-4-blocking monoclonal antibody, as monotherapy showed promising results in phase II and III studies among metastatic melanoma patients [98,99]. However, irAEs are quite frequent, which is consistent with the proposed mechanism of action of ipilimumab [100,101]. 

To improve therapeutic efficacy, immune checkpoint blockade has been combined with OVs (Table 2). VSV in combination with a CTLA-4 antibody was able to amplify a reproducible and statistically significant antitumor T cell immunologic response in murine mammary tumors, eliminating established macroscopic tumor implants [102]. It was also shown that the timing of anti-CTLA-4 mAb treatment was important since the effect depends on amplifying T cell responses [102]. NDV combined with CTLA-4 antibody was used to treat mouse B16 melanoma [16]. Intratumoral administration of NDV induced infiltration of activated CD4 and CD8 T cells in distant (non-virally injected) tumors in the absence of distant viral spread [16]. The inflammation made the tumor tissues susceptible to systemic CTLA-4 blockade therapy, leading to tumor rejection and resulted in increased survival rate in the mice treated with the combined therapy [16]. One systemic dose of vaccinia virus followed by three intra-peritoneal doses of anti-CTLA-4 antibody was used in a pre-clinical therapy in renal and colon adenocarcinoma mouse models, and the therapy showed a robust antitumoral response [103]. However, the induction of CD8 T and NK cells was only observed when the anti-CTLA-4 antibody was administered a few days after vaccinia virus injection [103]. Therapeutic potential of a systemically delivered VSV encoding tumor antigens c-Myc, HIF-2α, and Sox-10 in combination with anti-PD-1 or anti-CTLA-4 therapy has been shown in a pre-clinical glioma model [104]. The use of anti-PD-1 or anti-CTLA-4 alone prolonged the survival of mice [104]. However, the combined therapy with VSV-TAA showed an induction of the Th1 IFN-γ memory and TH17 response, and a significant increase in the survival [104]. The results demonstrated that VSV-TAA could induce a robust immune response when combined with ICIs [104].

PD-1 and its ligands, PD-L1 and PD-L2, are also emerging as promising targets for cancer therapy. PD-1, a transmembrane protein absents on resting naïve and memory T cells, plays an important role in regulating T cell activation [94]. It is transiently upregulated in activated T cells during antigen presentation [94]. PD-1 regulates T cell suppression through binding to PD-L1 or PD-L2 ligand, resulting in inhibition of T effector cell functions [94,105,106]. However, unlike CTLA-4, PD-1 can be expressed by other activated non-T-lymphocyte immune cell subsets, including B cells and NK cells [107]. The upregulation of PD-L1 has been observed on melanoma, lung and ovarian cancer, and glioma cells [17,108,109]. In vivo studies using PD-1, PD-L1, and PD-L2-knockout mice demonstrated a milder auto-immune phenotype compared to CTLA-4-knockout mice [106,110,111]. PD-1/PD-L1 blockers, such as pembrolizumab and nivolumab (anti-PD-1), and atezolizumab, durvalumab, and avelumab (anti-PD-L1), have shown activity in clinical trials, and are gaining approval for an expanding array of indications, including metastatic melanoma, advanced non-small-cell lung cancer, renal cell carcinoma, and classic Hodgkin’s lymphoma [112]. 

A double-stranded RNA virus reovirus (Reolysin, Oncolytics Biotech Inc., Calgary, AB, Canada) has been used as monotherapy or in combination with anti-PD-1 in pre-clinical and clinical studies [113,114]. In a melanoma mouse model, the combined therapy showed an induction of NK cell-mediated cytotoxicity and CD8-dependent antitumor T cell response as well as a reduction in Treg activity [113]. Another oncolytic virus used in combination with PD-1 blockade is Maraba rhabdovirus. Monotherapy of the virus has been shown to induce NK-mediated cytotoxicity, activate DC maturation, and enhance the production of pro-inflammatory cytokines and chemokines [115,116]. It is effective in several mouse tumor models [115] and is currently being tested in phase I and II clinical trials. A recent pre-clinical study used Maraba virus injected intratumorally or intravenously followed by intraperitoneal injections of both anti-PD1 and anti-CTLA-4 to treat triple-negative breast cancer [117]. Although virus treatment alone upregulated tumor cell PD-L1 expression and tumor-specific Tregs, the combination of the virus with ICIs demonstrated stronger inhibition of tumor growth than the individual therapies [117]. 

The combination of anti-CTLA-4 with T-VEC has been investigated in a Phase Ib clinical trial for the treatment of advanced melanoma where the intratumoral doses of T-VEC are followed by intravenous ipilimumab administration [118]. The combination had a tolerable safety profile, and showed greater efficacy than either T-VEC or ipilimumab monotherapy [118]. A phase II trial using the combination of Delta-24-RGD adenovirus and PD-1 has begun for patients with recurrent glioblastomas or gliosarcomas. One challenge that should be taken into consideration is that these clinical studies have been carried out in unresectable and advanced stages of cancer patients with slowed immune system. The potential of early stage treatment with more robust immune response should also be considered in order to prevent relapses.

Oncolytic viruses have also been engineered to encode antibodies against immune checkpoint receptors. The attenuated strains of measles virus (MV) encoding antibodies against CTLA-4 and PD-L1 (MV-aCTLA-4 and MV-aPD-L1) have been generated and tested in an immunocompetent murine model of malignant melanoma to evaluate the therapeutic efficacy of the virus [120]. The study showed that the viruses were equally efficient as parental MV in oncolytic efficacy against human tumors [120]. MV-aCTLA-4 enhanced antitumor immunity at early time points after treatment, while the effect of MV-aPD-L1 appeared at later phases of T cell activation in the periphery. This is consistent with CTLA-4 function, where immune responses within the lymphoid organs appeared in the early phase [120]. Contrastingly, PD-L1 signaling occurred at later phases of T cell activation in the periphery [120]. Systemic administration of anti-CTLA-4 or anti-PD-L1 together with intratumoral MV delayed tumor progression and prolonged survival, which was also observed with local, MV-mediated ICI expression [120]. Additionally, Ad5/3-Δ24aCTLA4, an oncolytic adenovirus expressing complete human mAb specific for CTLA-4, was evaluated in vitro and in vivo [119]. In this study, T cells of cancer patients, but not those of healthy donors, were activated by an anti-CTLA4 mAb produced by the virus [119]. High mAb concentrations were seen in tumors, compared to lower systemic levels of the antibody in mice [119].

Since T-VEC has been approved for melanoma patient treatment, the combination of this virus with ICIs has more advance in clinical testing than other OVs. Moreover, due to better safety profile of anti-PD-1 antibodies, there are more clinical trials for this ICI combined with OVs than for anti-CTLA-4 antibodies. We may expect encouraging results for this strategy in the near future. 

### 3.3. Oncolytic Viruses and Immune Co-Stimulation: Hit the “Gas” for T Cell Activation

Since the discovery of CD28 to prove the two-signal model of T cell activation, more and more molecules have been reported to have co-signaling function. The agonists for some of the co-stimulating receptors of the immunoglobulin (CD28, ICOS) and tumor necrosis factor (4-1BB, CD27, GITR, OX40) superfamily have been evaluated for cancer therapy [29,121,122,123]. Oncolytic viruses have been combined with 4-1BB, OX40 and ICOS co-stimulating pathways to enhance efficacy of virotherapy (Table 3). 

Co-stimulation through 4-1BB and OX40 promotes T cell survival through upregulation of anti-apoptotic factors and activation of AKT to promote cell cycling [29]. OX40 is also implicated with the upregulation of TRAF6, leading to activation of non-canonical NF-κB signaling and induction of IL-9 production in CD4+ T cells [128]. In immune-competent syngeneic mouse models of sarcoma and breast cancer, combining oncolytic vaccinia viruses (intratumoral) and anti-4-1BB agonist antibody (intraperitoneal) significantly reduced the growth of established subcutaneous tumors and the development of pulmonary metastatic lesions compared to either treatment alone [124]. The tumor inhibition was accompanied with an increased frequency of myeloid cells positive for CD11b+ and CD11c+ within the tumor draining lymph nodes, a heightened infiltration of CD8+ effector T and natural killer (NK) cells, and a maintained presence of neutrophils at the tumor site. [124]. Oncolytic vaccinia virus was also used to express 4-1BB ligand (4-1BBL) [125]. The recombinant virus rV-4-1BBL induced modest tumor regression in the poorly immunogenic B16 murine melanoma model [125]. Furthermore, in the context of lymphodepletion, rV-4-1BBL injection promoted MHC I expression, reduced antiviral antibody titers, promoted viral persistence, and rescued effector memory CD8+ T cells, leading to significant improvement of the therapeutic effectiveness of the oncolytic vector [125]. In the same murine melanoma model, an oncolytic adenovirus co-expressing IL-12 and 4-1BBL greatly enhanced the antitumor effect [72]. Co-administration of the virus with DCs elicited greater antitumor and anti-metastatic effects than either treatment alone [72]. The effect of the combination arms is associated with enhanced type-1 antitumor immune response and higher migratory abilities of DCs in tumors [72]. In addition, another oncolytic adenovirus LOAd703, which expresses both CD40L and 4-1BBL, was shown to be a potent immune activator that modulates the stroma to support antitumor responses when it was injected peritumorally in human pancreatic xenografts in nude mice [126].

Recently, we developed an oncolytic adenovirus Delta-24-RGDOX that expresses co-stimulator OX40 ligand (OX40L) [17]. Its receptor OX40 is expressed only in activated effector T cells [129], which means targeting this molecule spares the inactivated naïve T cells. The agonist antibody of OX40 has shown therapeutic effects in both mouse cancer models and late-stage human cancer patients [130,131]. In this new virus, the OX40L-expressing cassette replaced E3 region in the human adenovirus type 5 (Ad5) genome [17]. The therapeutic effect of this new construct was tested in syngeneic murine intracranial glioma models using immune competent mice. Compared to its predecessor Delta-24-RGD, Delta-24-RGDOX augmented the activation of lymphocytes by tumor cells and the expansion of the CD8+ T cells recognizing tumor-associated antigens, resulting in a tumor-specific immunity in immunocompetent mouse glioma models [17]. Consequently, Delta-24-RGDOX induced more potent anti-glioma activity [17]. Importantly, the virus synergized with intratumoral injections of anti-programmed death ligand 1 (PD-L1) antibody to reject gliomas in mice [17]. 

During CTLA-4 blockade therapy in cancer patients, a marked increase was found in the frequency of T cells expressing inducible co-stimulator (ICOS) in both tumor tissues and blood [132,133]. It was later found that ICOS pathway enhanced the efficacy of CTLA-4 blockade [122,123]. In addition to activation of PI3K-AKT pathway, ICOS also engaged the C-MAF pathway to induce secretion of IL-4 and IL-21 [29,134]. Its signaling is crucial for the induction of the transcriptional repressor BCL-6 which cooperates with C-MAF to promote the T_fh_ phenotype [135]. Lately, a recombinant NDV expressing ICOS ligand (NDV-ICOSL) was tested in the bilateral flank murine tumor models in immune competent mouse [127]. Intratumoral administration of NDV-ICOSL caused an enhanced infiltration of activated T cells in both virus-injected and distant tumors; importantly, treatment with NDV-ICOSL results in effective rejection of both tumors when used in combination with systemic CTLA-4 blockade [127].

Currently, compared to the other two strategies, this strategy is less-developed and is still at pre-clinical stage. We believe that the OVs armed with immune co-stimulator are worthy to be tested clinically due to the promising pre-clinical results and more tumor-specific immune stimulation.

## 4. Conclusions and Perspectives

For the last decade, the field of oncolytic virotherapy has experienced a rapid progress. The paradigm has changed from oncolysis to virus-mediated anti-tumor immunity. With the approval of T-VEC for intratumoral therapy of non-resectable metastatic melanoma, oncolytic virotherapy finally starts to deliver its promise as an alternative therapy for cancers that are refractory to traditional chemotherapy, radiotherapy, targeted therapy, and immunotherapy. It provides versatile platforms for designing and optimizing targeted molecular anticancer agents to execute cancer-selective killing through multiplex mechanisms, reducing the chance for cancer cells to develop resistance. 

Although T-VEC has gained approval for treatment in advanced melanoma, it doesn’t mean it will be a one-fits-all cancer virotherapy. Currently, OVs from various viral species are under clinical evaluation, including herpesvirus, adenovirus, measles virus, vaccinia virus, reovirus, poliovirus, coxsackievirus, VSV, parvovirus, and retrovirus [136]. Due to the complexity of each viral species, the researchers are usually experts on one virus but not on the others. There is a lack of knowledge in comparing the effect of individual viruses. Thus, it is hard to conclude which virus is advantageous over others for cancer therapy. In addition, different mechanisms of cancer selectivity for each OV also determine the diversified feasibility of OVs in various malignancies. However, when several OVs are suitable for a certain type of cancer, it may be more effective to choose a virus with low seroprevalence in the patients. Moreover, to avoid antiviral immunity developed during virotherapy to dampen the efficacy of the treatment, sequential use of immunologically non-cross-reactive multiple viruses may be adopted [137]. Therefore, before it is proven that one oncolytic viral platform is efficacious in all tumor types, the diversity of virotherapy will continue. Meanwhile, as our knowledge in virology and cancer biology expands, novel virotherapeutic strategies may appear as well. In a recent report, limited clinical data indicate that adenovirus and HSV have demonstrated better safety profile than reovirus and NDV with respect to virus-mediated adverse events in cancer patients [138]. With accumulating data from clinical testing, we should expect more statistically meaningful comparison of the safety and efficacy of different OVs in cancer patients. 

The in situ autovaccination of OVs has shown systemic effect on metastatic tumors. But there are still questions to be answered before we can further improve the efficacy to benefit more cancer patients. The disseminated metastatic tumors may already have well established immunosuppressive tumor microenvironment and/or include cancer cells differentiated from the ones in the virally treated tumor, and thus can become non-responsive to the anti-tumor immunity mediated by the intratumoral viral injection at one or limited tumor sites. Will systemically delivered OVs be able to induce in situ vaccination at each lesion site, resulting in more efficacious and sustainable antitumor immunity? To achieve this, OVs need to avoid rapid clearance from the circulation before they reach the tumor sites. One strategy is to circumvent preexisting immunity through choosing OVs with low seroprevalence, such as VSV [137], or modifying OVs to make them less immunogenic and recognizable by pre-existing immunity and/or redirected to the tumor sites [139,140,141]. Another strategy is to use cellular carriers to shield OVs from the adverse conditions in the blood stream. CTLs as well as other cell types, such as dendritic cells and mesenchymal stem cells, have been used as OV carriers [142,143]. However, before this “Trojan Horse” strategy can be efficiently utilized in clinic, several issues need to be addressed. The first is the tumor homing efficiency and specificity. The second is the cytotoxicity of OVs toward the carriers. The third is carrier cell engraftment efficiency.

Currently, we are excited to witness OVs moving from the benchtop to the bedside of cancer patients. There is still a lot to expect in the improvement of the efficacy of OVs by themselves or through combination with other therapies to increase anti-tumor immunity. Meanwhile, cautions should always be given to the safety of virotherapy through confining the viral effect only on tumors as much as possible. Therefore, optimizing virus-mediated in situ autovaccination to improve anti-tumor immunity is a promising strategy for this purpose.

## Figures and Tables

**Table 1:** Cytokine-armed OVs under clinical investigation.

Cytokine	Virus	Modification in Viral Genome	Tested Disease	Route of Administration	Clinical Status
GM-CSF	Human adenovirus 5 (ONCOS-102)	24-bp deletion in E1A; modified fiber with a serotype 3 knob	solid tumors refractory to available treatments	Intratumoral and intravenous	Phase I [36]
	Human adenovirus 5 (CG0070)	E2F-1 promoter /E1A gene, human GM-CSF insertion	Non-muscle invasive bladder cancer after BCG failure	Bladder instillation	Phase II/III [37,38]
	HSV-1 (T-VEC)	Deletion of ICP34.5, ICP47, human GM-CSF insertion	Unresected stage IIIB/C to IV melanoma with various metastasis	Intratumoral	Approved in the USA and Europe [39,40,41,42]
	Vaccinia virus (JX-594)	Thymidine kinase, human GM-CSF, lacZ insertion	Various cancers in adult and pediatric patients	Intravenous	Phase III trial [43,44,45,46,47]
IL-12	Human adenovirus 5 (Ad5-yCD/mutTKSR39rep-hIL12)	IL-12, yeast cytosine deaminase (CD), TKSR39 (thymidine kinase mutant) insertions	Non-small cell lung carcinoma (NSCLC) Prostate cancer	Intratumoral	Phase I (NSCLC) Phase II (prostate cancer)
	HSV-1 (M032)	Deletion of ICP34.5, IL-12 insertion	Recurrent/Progressive Glioblastoma Multiforme, Anaplastic Astrocytoma, Gliosarcoma	Intracerebral	Phase I [48]
IL-2	Vaccinia virus (VV-IL-2)	Deletion of thymidine kinase, insertion of IL-2	malignant mesothelioma	Intratumoral	Small pilot study with six patients [49]

**Table 2:** Combinational therapies with ICIs and OVs.

Antibodies	Virus	Modification in Viral Genome	Tested Disease	Route of Administration	Clinical Status
Anti-CTLA-4	VSV		Breast cancer	Intraperitoneally	Pre-clinical [102]
	NDV		Colon carcinoma and melanoma	Intratumoral dose of OV followed by intraperitoneal ICIs	Pre-clinical [16]
	HSV-1 (T-VEC)	Deletion of ICP34.5, ICP47, human GM-CSF insertion	Malignant melanoma	Intratumoral dose of OV followed by intravenous ICIs	Phase II [118]
	Human adenovirus 5 (Ad5/3-Delta24aCTLA4)	24-bp deletion in E1A; modified fiber with a serotype 3 knob; anti-CTLA-4 mAb insertion	Advanced solid tumors	Subcutaneous dose of OV followed by intraperitoneal ICIs	Pre-clinical [119]
Anti-CTLA-4 + anti-CD25	Vaccinia virus		Renal adenocarcinoma	Intravenous dose of OV followed by intraperitoneal ICIs	Pre-clinical [103]
Anti-CTLA-4 + anti-PD-1	VSV (VSV-HIF-2a, VSV-Sox-10, VSV-c-Myc)	c-Myc, HIF-2α, and Sox-10 insertion	Glioma	Intravenous dose of OV followed by intracranial ICIs	Pre-clinical [104]
	Maraba virus MG1		Triple-negative breast cancer	Intratumoral or intravenous dose of OV followed by intraperitoneal ICIs	Pre-clinical [117]
Anti-CTLA-4 or anti-PD-L1	Measles virus (MV-aCTLA-4, MV-aPD-L1)	Anti-CTLA-4 p4F10-γ1 or anti-PD-L1 mAb insertion	Melanoma	Intratumoral injection of OV	Pre-clinical [120]
Anti-PD-1	Reovirus		Melanoma	Intratumoral dose of OV followed by systemic ICIs	Pre-clinical [113]
	HSV-1 (T-VEC)	Deletion of ICP34.5, ICP47, human GM-CSF insertion	Unresectable Stage IIIB to IVM1c Melanoma	Intratumoral dose of OV followed by intravenous ICIs	Phase Ib/3
	HSV-1 (T-VEC)	Deletion of ICP34.5, ICP47, human GM-CSF insertion	Head and neck squamous cell carcinoma	Intratumoral dose of OV followed by intravenous ICIs	Phase Ib/3
	Human adenovirus 5 (DNX-2401)	24-bp deletion in E1A, RGD-4C motif insertion in fiber	Recurrent glioblastoma or gliosarcoma	Intratumoral dose of OV followed by intravenous ICIs	Phase II
	Human adenovirus 5 (ONCOS-102)	Insertion of human GM-CSF	Advanced or Unresectable Melanoma	Intratumoral dose of OV followed by intravenous ICIs	Phase I
	Maraba virus (MG1-MAGEA3)	Insertion of human melanoma antigen A3 (MAGE-A3)	Non-Small Cell Lung Cancer	Intratumoral dose of OV followed by intravenous ICIs	Phase I/II
	Human adenovirus 5 (ADV/HSV-tk)	Insertion of herpes simplex virus thymidine kinase (HSV-tk)	Metastatic triple negative breast cancer and metastatic non-small cell lung cancer	Intratumoral dose of OV followed by intravenous ICIs	Phase II

**Table 3:** Combinational therapies including immune co-stimulatory agents and OVs.

Virus	Modification in Viral Genome	Tested Disease	Route of Administration	Clinical Status
Vaccinia virus		Sarcoma and breast cancer	OV: intratumoral; anti-4-1BB: intraperitoneal	Pre-clinical [124]
Vaccinia virus (rV-4-1BBL)	Insertion of 4-1BBL	Melanoma	Intratumoral	Pre-clinical [125]
Human adenovirus 5 (Ad-ΔB7/IL-12/4-1BBL)	Insertion of IL-12 and 4-1BB	Melanoma	Intratumoral	Pre-clinical [72]
Human adenovirus 5 (LOAd703)	Co-insertion of CD40L and 4-1BBL	Human pancreatic xenografts in nude mice	Peritumoral injection	Pre-clinical [126]
Human adenovirus 5 (Delta-24-RGDOX)	24-bp deletion in E1A, RGD-4C motif insertion in fiber, insertion of OX40L	Glioma	OV: intratumoral; anti-PD-L1: intratumoral	Pre-clinical [124]
NDV (NDV-ICOSL)	Insertion of ICOSL	Melanoma	OV: Intratumoral; anti-CTLA-4: Intraperitoneal	Pre-clinical [127]
